# A symmetric version of the Euler equations by using Generalized Bernoulli Method

**DOI:** 10.1016/j.heliyon.2023.e16947

**Published:** 2023-06-07

**Authors:** U. Filobello-Nino, H. Vazquez-Leal, J. Huerta-Chua, D. Mayorga-Cruz, R. Lopez-Leal, R.A. Callejas Molina, M.A. Sandoval-Hernandez

**Affiliations:** a Facultad de Instrumentación Electrónica, Universidad Veracruzana, Cto. Gonzalo Aguirre Beltrán S/N, 91000 Xalapa, Veracruz, Mexico; b Consejo Veracruzano de Investigación Científica y Desarrollo Tecnológico (COVEICYDET), Av Rafael Murillo Vidal No. 1735, Cuauhtémoc, 91069 Xalapa, Veracruz, Mexico; c Instituto Tecnológico Superior de Poza Rica, Tecnológico Nacional de México, Luis Donaldo Colosio Murrieta S/N, Arroyo del Maíz, 93230 Poza Rica, Veracruz, Mexico; d Centro de Investigación en Ingeniería y Ciencias Aplicadas, CIICAP, Universidad Autónoma del Estado de Morelos, 62209 Cuernavaca, Morelos, Mexico; e Posgrado de Ciencias en la Ingeniería, Instituto Tecnológico de Celaya, Tecnológico Nacional de México, Antonio García Cubas Pte. 600, 38010 Celaya, Guanajuato, Mexico; f CBTis 190 DGETI. Av 15, Venustiano Carranza, Carranza 2da Sección, 94297 Boca del Río, Veracruz, Mexico

**Keywords:** Generalized Bernoulli Method, Variational problems, Euler equations, Ordinary differential equations, Isoperimetric problems

## Abstract

The aim of this article is to show a way to extend the usefulness of the Generalized Bernoulli Method (GBM) with the purpose to apply it for the case of variational problems with functionals that depend explicitly of all the variables. Moreover, after expressing the Euler equations in terms of this extension of GBM, we will see that the resulting equations acquire a symmetric form, which is not shared by the known Euler equations. We will see that this symmetry is useful because it allows us to recall these equations with ease. The presentation of three examples shows that by applying GBM, the Euler equations are obtained just as well as it does the known Euler formalism but with much less effort, which makes GBM ideal for practical applications. In fact, given a variational problem, GBM establishes the corresponding Euler equations by means of a systematic procedure, which is easy to recall, based in both elementary calculus and algebra without having to memorize the known formulas. Finally, in order to extend the practical applications of the proposed method, this work will employ GBM with the purpose to apply it for the case of solving isoperimetric problems.

## 1 Introduction

Calculus of variations is a mathematical discipline whose goal is the maximization or minimization of functionals [1], [2]. Functionals consist in a correspondence rule which associate functions to real numbers and are frequently expressed as definite integrals.

As it is well known, calculus of variations is relevant from several points of view; thus, for instance, it is known that the laws of physics are obtained from a variational principle, in such a way, that a functional reaches an extreme value with the end to obtain the laws that describe a physical process [3]. Among some relevant examples, we can mention the very known conservation laws of energy and momentum, Hamilton's principle, the Fermat principle, the Klein-Gordon equation.

In the same way, in quantum physics, Schrödinger used calculus of variations with the end to find his wave equation; other relevant example, is the Hartree-Fock variational method applied in order to describe atoms with many electrons, among many other examples.

Although, historically it is accepted that the variational calculus arose with the brachistochrone problem originally proposed by Bernoulli [4], [5], it was Leonhard Euler whom established it on general theoretical fundaments.

This work proposes two novelties to approach variational problems without resorting to Euler equations. Unlike of the previous works about GBM [6], [7], this article has the purpose to generalize the use of GBM for the case of variational problems which explicitly depend on all variables. The second novelty introduced by this article is concerned with the application of the proposed method to isoperimetric problems. As a matter of fact, we will see that GBM let us to obtain the differential equation for these problems with restrictions in a systematic way which eases the study of this topic.

Next, we mention some relevant methods proposed with the end to find solutions for variational problems. Reference [8] employed He's brackets and Ritz method in order to approach the finned tube heat exchanger used in hydride hydrogen storage system; while the case of variable two-end problems for the case of determining the unknown shape of oscillating airfoils in compressible 2d unsteady flow is considered in [9]. The inverse problem of the variational principle which is concerned with the issue to substitute the Lagrange multiplier method, is analysed in [10], [11], while the application of variational principles for the case of nonlinear partial differential equations with variable coefficients is considered in [12] among others.

This article is organized as follows. Section 2 provides the rudiments of variational calculus required for this work. Section 3 presents the basic elements of generalized Bernoulli method (GBM) studied so far. Section 4 deals with the original results of this paper. Section 5 applies the proposed methodology in order to solve three examples. Section 6 emphasizes the applications of the proposed method for the issues considered for this article. Finally, Section 7 provides the conclusions of the main aspects considered in this article, as well as the proposal for expanding the GBM even more for future works.

## 2 Basic rudiments of variational calculus

In what follows, we will be interested in the problem of extremizing functionals such as

(1) $$S[y]=\int_{x_{1}}^{x_{2}}f(x,y,y^{\prime })dx,$$

where we assume that the endpoints are fixed.

In accordance with variational calculus, it is assumed that $f(x,y,y^{\prime })$ is a function with continuous partial derivatives of second order with respect to *x*, *y*, and $y^{\prime }$ [3], the aim is determining a function y(x), that satisfies the boundary conditions; and maximizes or minimizes (1). A basic problem is to find the curve of shortest length joining two points. In this example, (1) becomes

(2) $$S[y]=\int_{x_{1}}^{x_{2}}\sqrt{1+{y^{\prime }}^{2}}dx,$$

i.e.

(3) $$f(x,y,y^{\prime })=\sqrt{1+{y^{\prime }}^{2}}.$$

The answer for the problem expressed by (2) and (3) is, of course, the straight line between the given points. Nevertheless, there exist problems by which the answer is complicated; for instance, the Brachistochrone problem which consists in determining a vertical curve, that joins two fixed points with no friction, through which a particle slides in the shortest time [2], [3], [6]. In this case, it is required to minimize

(4) $$S[y]=\int_{x_{1}}^{x_{2}}\frac{\sqrt{1+{y^{\prime }}^{2}}}{\sqrt{2gy}}dx,$$

where *g* denotes the acceleration of gravity and the function *f* for this case adopts the following form

(5) $$f(x,y,y^{\prime })=\frac{\sqrt{1+{y^{\prime }}^{2}}}{\sqrt{2gy}},$$

such as it was mentioned, the Bernoulli's solution for the problem given by (4) and (5) is particularly relevant for the proposal of GBM [6]. A systematic way to find a function which extremizes an integral of the form (1) is by utilizing the Euler equation [1], [2], [3].

(6) $$\frac{d}{dx}\left(\frac{\partial f}{\partial y^{\prime }}\right)-\left(\frac{\partial f}{\partial y}\right)=0.$$

Many times (6) is nonlinear and difficult to solve, nevertheless there have been found both exact [2], [3], [7], [13] and approximate [7], [13] solutions for variational problems.

If the variable *y* does not explicitly appear in function *f*, (6) adopts the following form [1], [2], [3]

(7) $$\frac{d}{dx}\left(\frac{\partial f}{\partial y^{\prime }}\right)=0,$$

and from (7) we get

(8) $$\frac{\partial f}{\partial y^{\prime }}=c,$$

where *c* is a constant of integration.

On the other hand, if *f* does not depend explicitly on *x*, then it is possible to show that (6) is expressed as:

(9) $$y^{\prime }\frac{\partial f}{\partial y^{\prime }}-f=k,$$

for some integration constant *k* [2], [3].

As a matter of fact, if *f* depends explicitly on *x*, then it is possible to express Euler equation (6) in the following relevant alternative form:

(10) $$\frac{d}{dx}\left(y^{\prime }\frac{\partial f}{\partial y^{\prime }}-f\right)=-\frac{\partial f}{\partial x}.$$

### 2.1 Isoperimetric constraints

A legend says that when Queen Dido fled from her brother Pygmalion along the north African coast, she arrived at the site that Tunisia currently occupies. There, she asked Jarbas for asylum and a place to live; he proposed her to keep an extension of land that she could cover with an ox hide; therefore, she cut the skin into strips which she joined at the ends in such a way that she planned the largest area with the strips keeping the perimeter fixed. It is said that somehow she found the correct answer, a circumference.

Mathematically, an isoperimetric problem is established in the following terms:

We consider the problem of extremizing a functional of the form (1), subjected to boundary conditions $y(x_{1})=a$, $y(x_{2})=b$, with the constraint condition

(11) $$\int_{x_{1}}^{x_{2}}g(x,y,y^{\prime })dx=k,$$

where *k* is a constant.

Integral conditions like (11) are denoted as isoperimetric constraints.

In accordance with variational theory, the above problem is solved by introducing a Lagrange multiplier *λ* such that the extremals of (1) subjected to (11) are determined from the extremals for the integral

(12) $$\int_{x_{1}}^{x_{2}}\left[f(x,y,y^{\prime })+\lambda g(x,y,y^{\prime })\right]dx.$$

The Euler equation for (12) is expressed as [2], [3]:

(13) $$\frac{d}{dx}\left(\frac{\partial f}{\partial y^{\prime }}\right)-\frac{\partial f}{\partial y}=Q,$$

where

(14) $$Q=\lambda \left[\frac{\partial g}{\partial y}-\frac{d}{dx}\left(\frac{\partial g}{\partial y^{\prime }}\right)\right],$$

for the case of several constraints like (11), it is also applied the Euler equation (13) but now *Q* is expressed as a sum of terms similar to the right side of (14).

Another manner to express the Euler equation (13) is in terms of a function *F*, that obeys (6) and given by

(15) $$F(x,y,y^{\prime })=f(x,y,y^{\prime })+\lambda g(x,y,y^{\prime }).$$

## 3 Basic elements of Generalized Bernoulli Method

This section presents the rudiments of GBM introduced in [6], [7] to review how to directly use it to write the Euler equations when one of the variables does not explicitly appear in the functional (cyclic variable) by using just both elementary algebra and calculus. To begin, we will assume functionals of the kind [6], [7].

(16) $$\int_{x_{1}}^{x_{2}}f(y,y^{\prime })dx.$$

The steps for GBM are:

1. We begin writing the derivatives and the differential *x*, in the integrand of (16), in terms of increments *δx* and *δy*.
2. Next, we differentiate with respect to *δx* the result deduced in the previous step, rewriting what results from this derivative in terms δy/δx quotient and equating to a constant.
3. Finally, applying the limit δx→0 in order to obtain the Euler equation.

We point out that while [6] showed that GBM works by solving several case studies, [7] introduced a more general argument as follows. We will derive (9) by using GBM (see [7]); for this purpose, we begin considering the integrand from (16) in terms of increments

(17) $$f\left(y,\frac{\delta y}{\delta x}\right)\delta x,$$

where we adopt the notation for increments proposed in [6]. Given that (17) does not explicitly depend on *x*, then in accordance with GBM, we will differentiate (17) with respect to *δx* and we will conclude this procedure equating the result to a constant.

(18) $$\frac{df\left(y,\frac{\delta y}{\delta x}\right)\delta x}{d(\delta x)}=f\left(y,\frac{\delta y}{\delta x}\right)+\delta x\left(-\frac{\delta y}{{\left(\delta x\right)}^{2}}\right)f_{y^{\prime }}\left(y,\frac{\delta y}{\delta x}\right),$$

where the last term of (18) results from the application of the chain's rule and, for the sake of simplicity, we will adopt from the beginning, the notation $f_{y^{\prime }}$ instead of $f_{\frac{\delta y}{\delta x}}$ to denote the derivative of *f* with respect to $\frac{\delta y}{\delta x}$, (it is worth mentioning that later, the limit δx→0 will be considered and therefore, $\lim_{\delta x\to 0}\frac{\delta y}{\delta x}=y^{\prime }$). This simplification will be adopted in other similar mathematical processes along this work. From (18) we obtain

(19) $$\frac{df\left(y,\frac{\delta y}{\delta x}\right)\delta x}{d(\delta x)}=f\left(y,\frac{\delta y}{\delta x}\right)-\left(\frac{\delta y}{\delta x}\right)f_{y^{\prime }}\left(y,\frac{\delta y}{\delta x}\right).$$

After regarding the limit as δx→0, (19) becomes in

(20) $$f\left(y,\frac{\delta y}{\delta x}\right)-\left(\frac{\delta y}{\delta x}\right)f_{y^{\prime }}\left(y,\frac{\delta y}{\delta x}\right)\to f\left(y,y^{\prime }\right)-y^{\prime }f_{y^{\prime }}(y,y^{\prime }).$$

From (20), we obtain the Euler equation (9) after equating to a constant the expression that follows the arrow.

Next, we will assume functionals of the form

(21) $$\int_{x_{1}}^{x_{2}}f(x,y^{\prime })dx.$$

Expressing the integrand of (21) in terms of increments

(22) $$f\left(x,\frac{\delta y}{\delta x}\right)\delta x.$$

Given that expression (22) is not an explicit function of *y*, then differentiating (22) with respect to *δy* [6], [7]

(23) $$\frac{df\left(x,\frac{\delta y}{\delta x}\right)\delta x}{d(\delta y)}=\delta x\left(\frac{1}{\left(\delta x\right)}\right)f_{y^{\prime }}\left(x,\frac{\delta y}{\delta x}\right),$$

and from (23)

(24) $$\frac{df\left(x,\frac{\delta y}{\delta x}\right)\delta x}{d(\delta y)}=f_{y^{\prime }}\left(x,\frac{\delta y}{\delta x}\right).$$

Taking the limit as δx→0, the right hand side of (24) adopts the form.

(25) $$f_{y^{\prime }}\left(x,\frac{\delta y}{\delta x}\right)\to f_{y^{\prime }}(x,y^{\prime }).$$

In accordance with the proposed method, we equate to a constant the expression after the arrow in (25), assuming that *f* is not explicitly expressed in terms of *y*. This last equation is equivalent to Euler equation (8).

## 4 Contributions of this work

We will inquire the GBM version of Euler equation (10), therefore, we will assume integrals of the form (1).

Next, we express the integrand of (1) in terms of increments *δx* and *δy* (substituting differential *x* as *δx*) and we will differentiate the expression resulting from the generalized Bernoulli procedure with respect to *δx*, rewriting the resulting expression in terms of δy/δx ratio. As a matter of fact, we will analyse the effect of including the *x* variable in the integrand of (1).

For it, we begin expressing the integrand from (1) in terms of increments

(26) $$f\left(x,y,\frac{\delta y}{\delta x}\right)\delta x,$$

where we have introduced the notation for increments adopted in [6].

**Figure 1:**
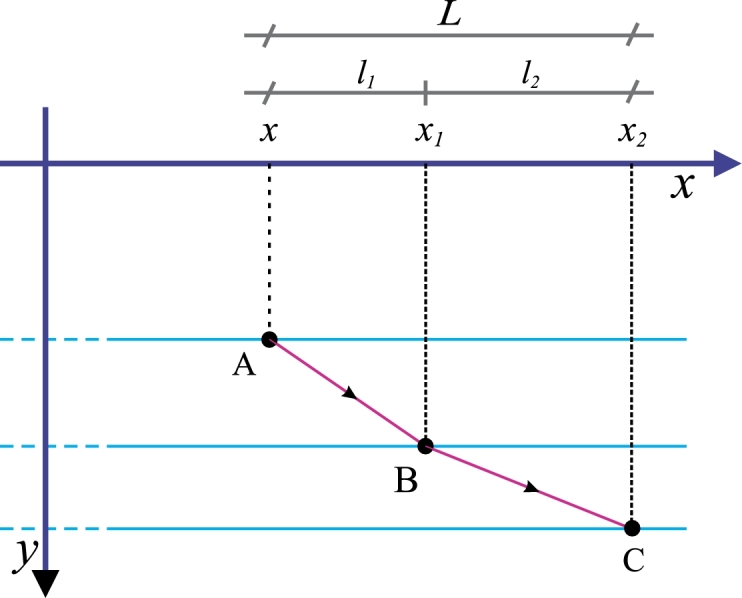
Ray of light entering a second medium.

Fig. 1 focusses at a particular region where a “light beam” crosses from one medium to another (from A to B and from B to C) [3], [6]. In accordance with GBM, the total time spent travelling from the point A to the point C is given by:

(27) $$T=f\left(x,y,\frac{\delta y}{\delta x}\right)\delta x+f\left(x+\delta x,y+\delta y,\frac{\delta y_{1}}{\delta x_{1}}\right)\delta x_{1}.$$

From the same figure we note that:

(28) $$(x_{1}-x)+(x_{2}-x_{1})=L,$$

from (28)

(29) $$l_{1}+l_{2}=L$$

therefore, from (29) we get

(30) $$\delta x_{1}=-\delta x.$$

We will optimize *T* differentiating (27) respect to *δx*, and equating the result to zero:

(31) $$\frac{dT}{d\left(\delta x\right)}=f\left(x,y,\frac{\delta y}{\delta x}\right)+f_{y^{\prime }}\left(x,y,\frac{\delta y}{\delta x}\right)\left(\frac{-\delta y}{\delta x}\right)+f\left(x+\delta x,y+\delta y,\frac{\delta y_{1}}{\delta x_{1}}\right)\frac{\delta x_{1}}{\delta x}+f_{x}\left(x+\delta x,y+\delta y,\frac{\delta y_{1}}{\delta x_{1}}\right)\delta x_{1}+f_{y_{1}^{\prime }}\left(x+\delta x,y+\delta y,\frac{\delta y_{1}}{\delta x_{1}}\right)\left(-\frac{\delta y_{1}}{\delta x_{1}^{2}}\right)\frac{d\delta x_{1}}{d\delta x}\delta x_{1}=0.$$

After employing (30) we express (31) as

(32) $$\frac{dT}{d\left(\delta x\right)}=f\left(x,y,\frac{\delta y}{\delta x}\right)-f_{y^{\prime }}\left(x,y,\frac{\delta y}{\delta x}\right)\left(\frac{\delta y}{\delta x}\right)-f\left(x+\delta x,y+\delta y,\frac{\delta y_{1}}{\delta x_{1}}\right)+f_{x}\left(x+\delta x,y+\delta y,\frac{\delta y_{1}}{\delta x_{1}}\right)\delta x_{1}+f_{y_{1}^{\prime }}\left(x+\delta x,y+\delta y,\frac{\delta y_{1}}{\delta x_{1}}\right)\left(\frac{\delta y_{1}}{\delta x_{1}}\right)=0.$$

After performing a little bit of algebra, we express (32) as

(33) $$\left(f\left(x,y,\frac{\delta y}{\delta x}\right)-f\left(x+\delta x,y+\delta y,\frac{\delta y_{1}}{\delta x_{1}}\right)\right)+\left(f_{y_{1}^{\prime }}\left(x+\delta x,y+\delta y,\frac{\delta y_{1}}{\delta x_{1}}\right)\left(\frac{\delta y_{1}}{\delta x_{1}}\right)-f_{y^{\prime }}\left(x,y,\frac{\delta y}{\delta x}\right)\left(\frac{\delta y}{\delta x}\right)\right)+f_{x}\left(x+\delta x,y+\delta y,\frac{\delta y_{1}}{\delta x_{1}}\right)\delta x_{1}=0.$$

Dividing (33) by $\delta x_{1}$ and considering that

(34) $$\frac{\delta y_{1}}{\delta x_{1}}=\frac{\delta y}{\delta x}(x+\delta x,y+\delta y),$$

then, from (34) we get

(35) $$\frac{-(f\left(x+\delta x,y+\delta y,\frac{\delta y_{1}}{\delta x_{1}}\right)-f\left(x,y,\frac{\delta y}{\delta x}\right))+(f_{y_{1}^{\prime }}\left(x+\delta x,y+\delta y,\frac{\delta y_{1}}{\delta x_{1}}\right)\left(\frac{\delta y_{1}}{\delta x_{1}}\right)-f_{y^{\prime }}\left(x,y,\frac{\delta y}{\delta x}\right)\left(\frac{\delta y}{\delta x}\right))}{\delta x_{1}}+f_{x}\left(x_{1}+\delta x,y_{1}+\delta y,\frac{\delta y_{1}}{\delta x_{1}}\right)=0.$$

Following the procedure explained in [3], [6], we allow the layers become thinner and more numerous, thus the *x* increment tends to zero $\delta x_{1}\to 0$, in this limit (35) assumes the form

(36) $$\frac{d\left(y^{\prime }f_{y^{\prime }}\left(x,y,y^{\prime }\right)-f\left(x,y,y^{\prime }\right)\right)}{dx}+f_{x}\left(x,y,y^{\prime }\right)=0,$$

we note that (36) is the Euler equation (10). Therefore, the effect of differentiating with respect to *δx* including *x* variable in *f* is to obtain one of the Euler equations. On the other hand, it is clear from (9), (19) and (20) that (36) is expressed as

(37) $$-\frac{d}{dx}\left(\frac{df\left(x,y,\frac{\delta y}{\delta x}\right)\delta x}{d(\delta x)}\right)+f_{x}\left(x,y,y^{\prime }\right)=0,$$

where it is understood that before applying the derivative d/dx, we have to take the limit δx→0 inside the parenthesis; nevertheless, this procedure does not introduce practical difficulties, it only indicates the substitution δy/δx→dy/dx; also, it is clear that independently from deduction (26)-(37), the result (37) can be inferred from (10), (19) and (20). Equation (37) is one of the GBM equations sought. Reciprocally, the GBM equation, for the case of integrals (16), is deduced from (37) for $f_{x}=0$. For the sake of simplicity, we will express the other Euler equation (6) in terms of the GBM from (24), (25) and (6) we infer that

(38) $$\frac{d}{dx}\left(\frac{df\left(x,y,\frac{\delta y}{\delta x}\right)\delta x}{d(\delta y)}\right)-f_{y}\left(x,y,y^{\prime }\right)=0,$$

where again, it is also understood that before applying derivative d/dx, we have to take limit δx→0. Equation (38) is the second GBM equation. In the same way, we note that the equivalence of GBM and Euler formalism for integrals (21) is obtained from (38) for the case where $f_{y}=0$.

It is useful to compare the Euler equations (6) and (10) with the GBM version of Euler equations mentioned above; we note that equations (37) and (38) are more compact, and above all, they remain invariant after interchanging the derivatives with respect to *δx* and *δy*, as well as the partial derivatives with respect to *x* and *y*, respectively. Therefore, equations (37) and (38) are symmetric with respect to these exchanges.

In a nutshell, equations (37) and (38) are the proposed equations for this article for the solutions of variational problems that emanate from functionals of the form (1). From (37) and (38) it is clear that, given the symmetry of the proposed equations, it is possible to select any of them, essentially, with the same ease; therefore, if we choose (37), then we follow the steps given by (18)-(20) and the expression after the arrow is differentiated respect to *x* in accordance with (37); then we add to the above, the partial derivative of *f* respect to *x* and finally, this is equated to zero.

In a similar way, to use (38), we follow the steps (23)-(25) and the expression after the arrow is also differentiated with respect to *x*; now, to this result, it is subtracted the partial derivative of *f* respect to *y* and finally, it is equated to zero.

It is worth noticing that independently that we differentiate respect to *δx* or *δy*, what results is derived with respect to *x*, and the second term will be $f_{x}$ if previously we differentiate respect to *δx* or $-f_{y}$ if we choose (38) and we previously differentiate respect to *δy*. This procedure is clearly easier to remind than equations (6) and (10).

### 4.1 Application of Generalized Bernoulli Method to solve isoperimetric problems

We note from (15) that the presence of the Lagrange multiplier does not change the fact that *F* is a function of *x*, *y*, and $y^{\prime }$; therefore, the application of GBM for isoperimetric problems corresponds to equations (37) and (38) without performing any change. Additionally, we will see that the application of GBM provides the Euler equations faster and with less effort than Euler formalism (see Case studies Section).

## 5 Case studies

Next, we will employ GBM for the solution of three isoperimetric problems in order to show the practical convenience of this method.

### 5.1 Queen Dido's Isoperimetric Problem

**Figure 2:**
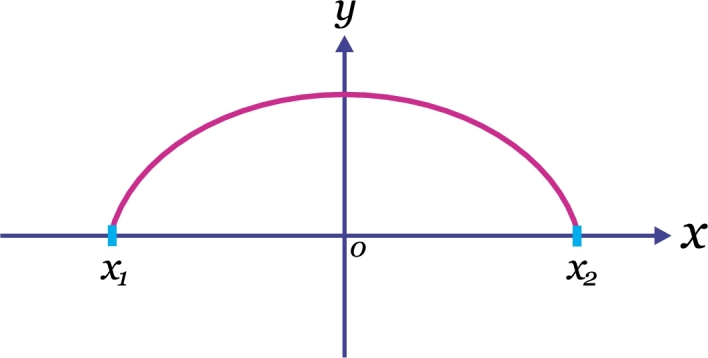
Determine the curve of length *l* in the upper half-plane passing through (*x*_1_,0) and (*x*_2_,0) that, together with the interval [*x*_1_,*x*_2_] encloses the maximum area.

Such as it was mentioned above, the Dido's Problem consists in finding the curve of fixed length *l* that joins two points which are lying above the *x*-axis, enclosing the maximum area between itself and the *x*-axis (see Fig. 2).

Mathematically, the problem consists in maximizing the integral

(39) $$\int_{-b}^{b}y(x)dx,$$

where (39) is subjected to the side condition

(40) $$\int_{-b}^{b}\sqrt{1+y^{\prime \;2}(x)}dx=l,$$

and for convenience, we have considered as end points, the points (−b,0) and (b,0) for some value *b* (we note that (40) is the mathematical expression for a curve of fixed length *l*).

In accordance with (15) we construct the function

(41) $$f=y+\lambda \sqrt{1+y^{\prime \;2}},$$

so that, the variational problem to solve is expressed in terms of the functional

(42) $$\int_{-b}^{b}\left(y(x)+\lambda \sqrt{1+y^{\prime \;2}(x)}\right)dx.$$

We will solve this problem using two manners.

**First**

We will take into account that (41) does not explicitly contain *x*, therefore (37) adopts the form [6], [7].

(43) $$\lim_{\delta x\to 0}\left(\frac{df\left(x,y,\frac{\delta y}{\delta x}\right)\delta x}{d(\delta x)}\right)=c_{1},$$

where *f* is given by (41).

Expressing the integrand of (42) (including *dx*) in terms of increments we obtain

(44) $$ydx+\lambda \sqrt{1+y^{\prime \;2}}dx\to y\delta x+\lambda \sqrt{\delta x^{2}+\delta y^{2}}.$$

In accordance with (43), we differentiate with respect to *δx* the increments expression to the right of the arrow of (44) to obtain

(45) $$y+\frac{\lambda }{\sqrt{1+{\left(\frac{\delta y}{\delta x}\right)}^{2}}}.$$

Taking the limit δx→0, in (45) and equating this result to a constant *c* we get the differential equation for Dido's problem

(46) $$y+\frac{\lambda }{\sqrt{1+y^{\prime \;2}}}=c.$$

Next, we will obtain the extremals from (46):

After a little algebra, it is possible to rewrite (46) as follows

(47) $$y^{\prime }=\frac{\sqrt{\lambda^{2}-{\left(y-c\right)}^{2}}}{y-c}.$$

After separating variables in (47) and integrating we get

(48) $${\left(x-c_{1}\right)}^{2}+{\left(y-c\right)}^{2}=\lambda^{2},$$

where $c_{1}$ is an integration constant, thus the extremals are circumferences.

**Second**

We will solve the above problem by using (38).

First, we will evaluate

(49) $$\lim_{\delta x\to 0}\left(\frac{df\left(x,y,\frac{\delta y}{\delta x}\right)\delta x}{d(\delta y)}\right),$$

differentiating the increments expression to the right of the arrow of (44) with respect to *δy* we obtain:

(50) $$\frac{\lambda \left(\delta y/\delta x\right)}{\sqrt{1+{\left(\frac{\delta y}{\delta x}\right)}^{2}}},$$

taking the limit δx→0 in (50), in accordance with (49), we get

(51) $$\frac{\lambda y^{\prime }}{\sqrt{1+y^{\prime \;2}}}.$$

The substitution of (51) into (38) yields in

(52) $$\frac{d}{dx}\left(\frac{\lambda y^{\prime }}{\sqrt{1+y^{\prime \;2}}}\right)=1,$$

where $f_{y}=1$ was obtained from (41).

After integrating (52) we obtain

(53) $$\frac{\lambda y^{\prime }}{\sqrt{1+y^{\prime \;2}}}=x+c,$$

next, we will integrate (53), with this purpose, it is possible rewriting it as follows

(54) $$y^{\prime }=\frac{x+c}{\sqrt{\lambda^{2}-{\left(x+c\right)}^{2}}},$$

after separating variables in (54) and integrating we get

(55) $${\left(x-c^{\prime }\right)}^{2}+{\left(y-c_{2}\right)}^{2}=\lambda^{2},$$

where $c^{\prime }$ and $c_{2}$ are constants.

From (48) and (55) we conclude that both procedures led to the same result, as it had to be. Thus, the maximum area is that enclosed by a semicircle of radius *λ* and the *x*-axis. In accordance with the assumptions mentioned in subsection 5.1, if the end points are (−b,0) and (b,0) then the centre of the circle is the origin (0,0) and λ=b. From the above, we deduce that the sought function is given by $y=\sqrt{b^{2}-x^{2}}$.

### 5.2 Find the form of a flexible non-extensible homogeneous rope of length *l* suspended at the points A and B by minimizing its potential energy

**Figure 3:**
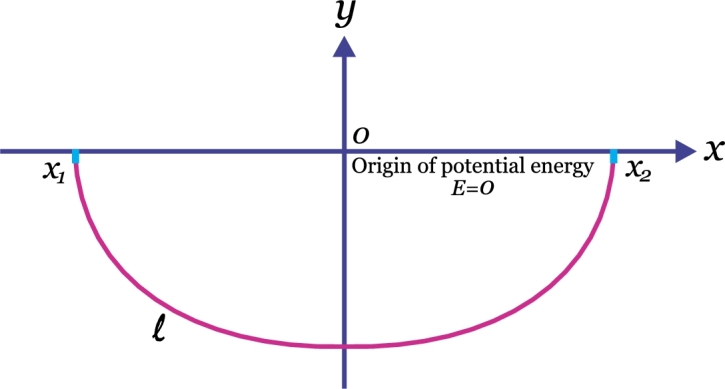
A heavy, uniform and flexible rope of length *l* hangs in equilibrium, under gravity from two fixed points.

We have to minimize the energy of the rope, given by [1], [3] (see Fig. 3)

(56) $$E=-\mu g\int_{x_{1}}^{x_{2}}y(x)\sqrt{1+y^{\prime \;2}(x)}dx,$$

subjected to boundary conditions $y(x_{1})=y_{a}$, $y(x_{2})=y_{b}$, where *g* is the gravity acceleration and *μ* is the density of the rope with the isoperimetric constraint

(57) $$l=\int_{x_{1}}^{x_{2}}\sqrt{1+y^{\prime \;2}(x)}dx.$$

In accordance with (12), with the purpose to minimize (56) subjected to (57) we propose the following functional

(58) $$e=\int_{x_{1}}^{x_{2}}\left(-\mu gy(x)\sqrt{1+y^{\prime \;2}(x)}+\lambda \sqrt{1+y^{\prime \;2}(x)}\right)dx,$$

thus, from (58), we define

(59) $$f=-\mu gy\sqrt{1+y^{\prime \;2}}+\lambda \sqrt{1+y^{\prime \;2}}.$$

From (59) the corresponding function in terms of increments is given by

(60) $$f=-\mu gy\sqrt{1+{\left(\frac{\delta y}{\delta x}\right)}^{2}}+\lambda \sqrt{1+{\left(\frac{\delta y}{\delta x}\right)}^{2}},$$

and (60) leads to

(61) $$f\delta x=-\mu gy\sqrt{\delta x^{2}+\delta y^{2}}+\lambda \sqrt{\delta x^{2}+\delta y^{2}}.$$

Given that *f* does not explicitly contain *x*, then we will differentiate (61) respect to *δx*, that is

(62) $$\frac{df\left(y,\frac{\delta y}{\delta x}\right)\delta x}{d(\delta x)},$$

and after considering the limit as δx→0 in (62).

Therefore:

(63) $$\frac{df\delta x}{d(\delta x)}=\frac{-\mu gy\delta x}{\sqrt{\delta x^{2}+\delta y^{2}}}+\lambda \frac{\delta x}{\sqrt{\delta x^{2}+\delta y^{2}}}.$$

After performing some algebra in (63)

(64) $$\frac{df\delta x}{d(\delta x)}=\frac{-\mu gy}{\sqrt{1+{\left(\frac{\delta y}{\delta x}\right)}^{2}}}+\frac{\lambda }{\sqrt{1+{\left(\frac{\delta y}{\delta x}\right)}^{2}}},$$

therefore, considering the limit δx→0 in (64) and equating this result to a constant:

(65) $$\frac{-\mu gy+\lambda }{\sqrt{1+y^{\prime \;2}}}=c^{\prime },$$

this is the differential equation to solve.

To complete the problem we will solve (65) for $y^{\prime }$

(66) $$y^{\prime }=\frac{\sqrt{{\left(\lambda -\mu gy\right)}^{2}-c}}{\sqrt{c}},$$

where $c=c^{\prime \;2}$, after separating the variables, we rewrite (66) as follows:

(67) $$\frac{dy}{\sqrt{{\left(\lambda -\mu gy\right)}^{2}-c}}=\frac{dx}{\sqrt{c}}.$$

Integrating (67) (by using the result)

(68) $$\int \frac{dy}{\sqrt{y^{2}-A^{2}}}=\ln\left[\frac{y+\sqrt{y^{2}-A^{2}}}{A}\right],$$

we get from (68) and a little algebra

(69) $$y=\frac{\lambda }{\mu g}-\frac{c_{3}}{\mu g}\cosh\left(\frac{-\mu gx-c_{2}}{c_{3}}\right),$$

for constants $c_{2}$ and $c_{3}$; therefore from (69), the solution is a family of catenaries [1].

As a matter of fact, if we assume, for the sake of simplicity, that the end points are of coordinates y(±b)=0, then it is possible to show that the function that minimizes the energy of the rope is given by $y=\frac{1}{A}[\cosh(Ax)-\cosh(Ab)]$, where *A* is a constant related with the length *l* (see (57)); also, we note that the catenary is the curve that joins two points and generates the minimum surface of revolution when it rotates around a coplanar axis with the points.

### 5.3 Comparison between Euler Formalism and Generalized Bernoulli Method with the end to obtain the differential equation for an Isoperimetric Variational Problem

With this purpose we propose the following integral

(70) $$\int_{x_{1}}^{x_{2}}y(x)y^{\prime }(x)\sqrt{1+y^{\prime \;2}(x)}dx,$$

subject to the side condition

(71) $$\int_{x_{1}}^{x_{2}}\sqrt{1+y^{\prime \;2}(x)}dx=l.$$

To solve the variational problem expressed by (70) and (71), we have to extremize the integral

(72) $$\int_{x_{1}}^{x_{2}}\left(y(x)y^{\prime }(x)\sqrt{1+y^{\prime \;2}(x)}+\lambda \sqrt{1+y^{\prime \;2}(x)}\right)dx,$$

we denote the integrand of (72) as

(73) $$f=yy^{\prime }\sqrt{1+y^{\prime \;2}}+\lambda \sqrt{1+y^{\prime \;2}}.$$

**Euler Formalism**

Since (73) does not contain *x* explicitly, then we will use the form (9) of Euler equation; where *f* is given by (73).

We will perform the procedure step by step

(74) $$y^{\prime }\frac{\partial f}{\partial y^{\prime }}=y\left(\frac{y^{\prime \;3}}{\sqrt{1+y^{\prime \;2}}}+y^{\prime }\sqrt{1+y^{\prime \;2}}\right)+\frac{\lambda y^{\prime \;2}}{\sqrt{1+y^{\prime \;2}}},$$

therefore

(75) $$y^{\prime }\frac{\partial f}{\partial y^{\prime }}-f=y\left(\frac{2y^{\prime \;3}+y^{\prime }}{\sqrt{1+y^{\prime \;2}}}\right)+\frac{\lambda y^{\prime \;2}}{\sqrt{1+y^{\prime \;2}}}-yy^{\prime }\sqrt{1+y^{\prime \;2}}-\lambda \sqrt{1+y^{\prime \;2}}.$$

Simplifying (75), after performing a cumbersome algebraic procedure

(76) $$y^{\prime }\frac{\partial f}{\partial y^{\prime }}-f=\frac{y\left(2y^{\prime \;3}+y^{\prime }\right)+\lambda y^{\prime \;2}-yy^{\prime }-yy^{\prime \;3}-\lambda -\lambda y^{\prime \;2}}{\sqrt{1+y^{\prime \;2}}},$$

or

(77) $$y^{\prime }\frac{\partial f}{\partial y^{\prime }}-f=\frac{yy^{\prime \;3}-\lambda }{\sqrt{1+y^{\prime \;2}}},$$

and equating (77) to a constant

(78) $$\frac{yy^{\prime \;3}-\lambda }{\sqrt{1+y^{\prime \;2}}}=k.$$

This is the sought differential equation, although we saved several steps between equations (74)-(78), intentionally we showed several algebraic steps with the purpose to expose a procedure rather cumbersome to get the result.

**GBM Formalism**

In accordance with GBM, we obtain the function in terms of increments:

(79) $$g=y\left(\frac{\delta y}{\delta x}\right)\sqrt{1+{\left(\frac{\delta y}{\delta x}\right)}^{2}}+\lambda \sqrt{1+{\left(\frac{\delta y}{\delta x}\right)}^{2}},$$

after a little bit of algebra we express (79) as

(80) $$g\delta x=y\delta y\sqrt{1+\delta y^{2}\delta x^{-2}}+\lambda \sqrt{\delta x^{2}+\delta y^{2}}.$$

Applying an ordinary derivative to (80) respect to *δx* we get

(81) $$\frac{dg\delta x}{d(\delta x)}=\frac{-y\delta y^{3}\delta x^{-3}}{\sqrt{1+\delta y^{2}\delta x^{-2}}}+\frac{\lambda \delta x}{\sqrt{\delta x^{2}+\delta y^{2}}},$$

rewriting (81) we get

(82) $$\frac{dg\delta x}{d(\delta x)}=\frac{-y{\left(\delta y/\delta x\right)}^{3}}{\sqrt{1+{\left(\frac{\delta y}{\delta x}\right)}^{2}}}+\frac{\lambda }{\sqrt{1+{\left(\frac{\delta y}{\delta x}\right)}^{2}}}.$$

Finally, considering the limit δx→0 in (82), and equating to a constant:

(83) $$\frac{yy^{\prime \;3}-\lambda }{\sqrt{1+y^{\prime \;2}}}=k^{\prime },$$

we note that (83) is the same result obtained in (78), but with much less effort. We emphasize also that we did not memorize any formula, just expressed the integrand in terms of increments and differentiate.

## 6 Discussion

The goal of this work is to widen the applications of GBM for the case of isoperimetric problems and above all to express the variational problems without cyclic variables in terms of the proposed method. Articles [6], [7] showed the usefulness of GBM for the case of integrals like (1) where one of the variables *x* or *y* is absent. In particular [7] exposed the way to get both, exact and analytical approximate solutions for certain variational problems with moving boundaries but without resorting to Euler formalism.

As a matter of fact, from articles [6], [7] we deduced that GBM is a method that provides the differential equation for some given variational problems making use only of elementary differentiations and basic algebra and this work not only emphasizes this point, but allows to write Euler equations (6) and (10) in a symmetric way.

We note that the known Euler equations are not symmetric because they do not pose the same mathematical form. Unlike those equations, (37) and (38) have an obvious symmetry, whereby, they are not difficult to remember; in fact, it is clear that it does not matter if the differentiation is with respect to *δx* or with respect to *δy*, the result is differentiated respect to *x* and then to this, it is subtracted the partial differentiation of *f* respect to *x* and *y*, respectively; we note that this procedure is systematic and involves only basic algebra and elementary differentiations.

Such as it was mentioned in [6], we remember that for the case of a cyclic variable, the above rule is still easier to remember: if for instance, *x* is absent in (1) then the differential equation is obtained from $\lim_{\delta x\to 0}\left(df\left(x,y,\frac{\delta y}{\delta x}\right)\delta x/d(\delta x)\right)=c_{1}$; in a nut shell, if *x* is absent then *f* is differentiated respect to *δx* and the result is equated to a constant, while something similar occurs if *y* is the absent variable. We noted that GBM performed well for the case of isoperimetric problems, following the procedure above mentioned in this article, therefore, we applied GBM for solving three relevant case studies.

The first of them, consisted in the solution for the Queen Dido's isoperimetric problem by using two procedures: the first one employed the fact that the function (41) does not contain *x* and therefore we used (43) with the purpose to get the differential equation (46) for the variational problem of Dido. After we obtained (52) by using (38) which is one of the GBM version of Euler equations proposed for this work. The solutions of (46) and (52) resulted in the same answer such as it had to occur, a circumference. On the other hand, the second example was concerned with the isoperimetric problem of finding the form of a flexible non-extensible homogeneous rope of length *l* suspended between two points A and B, with this purpose, we minimize the energy of the rope (56) with the constraint (57).

We differentiated the corresponding function in terms of increments (61) respect to *δx* given that it does not contain explicitly *x*; as consequence, we obtained equation (65) with little effort by following the simple rules of the proposed method which are easily remembered; finally, we completed the problem by solving (65) to determine that the extreme curves are a family of catenaries. The third case study consisted in finding the differential equation for an isoperimetric variational problem comparing the Euler formalism and GBM. Although this variational problem does not describe a known case, this example showed that the obtaining of the differential equation by using GBM is easier and straightforward in comparison with Euler formalism; moreover, the set of known Euler operations (74)-(78) is long and involves both cumbersome differentiations and algebraic steps; while, GBM steps (79)-(82) involved basic algebra and elementary differential calculus; in a sequence, many times GBM gets the differential equation at naked eye, which is of great importance from the practical point of view.

## 7 Conclusions

This work extended the usefulness of GBM with the end to show how to use it, in order to directly write the Euler equations with the end to extremize (1) by using a systematic procedure based on elementary calculus, just like it was reported in [6], [7]; this work also emphasized the practical importance of this method. For that purpose, GBM allowed us to write the variational differential equations following a systematic and elementary procedure with little effort, which is applicable without keeping in mind the known Euler formalism.

The above discussion is consequence of one of the main contributions of this work, this article obtained a symmetrical version for the known Euler equations and the importance of this generalization of the proposed method lies not only in the fact that the proposed equations are easier to remind; besides, from the theoretical point of view, this work showed the equivalence of the procedure that yielded in our symmetrical version and the corresponding variational procedure that allows to deduce the classical Euler equations [1], [2], [3]; therefore, at least for the case of variational problems involving integrals of the kind (1), both mathematical procedures are equivalents.

At the same time, in order to continue with the applications of GBM, this work proposed its application for isoperimetric problems with good results. As future work, we propose to extend the use of GBM for the case of functionals depending of several dependent variables and one independent variable, as well as for the case of functionals depending on double integrals.

## Funding statement

This research did not receive any specific grant from funding agencies in the public, commercial, or not-for-profit sectors.

## CRediT authorship contribution statement

U. Filobello-Nino; H. Vazquez-Leal: Conceived and designed the experiments; Performed the experiments; Analyzed and interpreted the data; Contributed reagents, materials, analysis tools or data; Wrote the paper.

J. Huerta Chua; D. Mayorga-Cruz; R. Lopez-Leal: Performed the experiments; Analyzed and interpreted the data; Contributed reagents, materials, analysis tools or data.

R.A. Callejas Molina; M.A. Sandoval-Hernandez: Performed the experiments; Analyzed and interpreted the data.

## Declaration of Competing Interest

The authors declare that they have no known competing financial interests or personal relationships that could have appeared to influence the work reported in this paper.

## Data Availability

Data included in article/supp. material/referenced in article.
